# BCG vaccination policy and preventive chloroquine usage: do they have an impact on COVID-19 pandemic?

**DOI:** 10.1038/s41419-020-2720-9

**Published:** 2020-07-08

**Authors:** Abhibhav Sharma, Saurabh Kumar Sharma, Yufang Shi, Enrico Bucci, Ernesto Carafoli, Gerry Melino, Arnab Bhattacherjee, Gobardhan Das

**Affiliations:** 1 School of Computer and System Sciences, Jawaharlal Nehru University, New Delhi, India; 2 The First Affiliated Hospital of Soochow University, State Key Laboratory of Radiation Medicine and Protection, Institutes for Translational Medicine, Soochow University Medical College, Suzhou, China; 3 Resis Srl, Samone, 10010 TO Italy; 4 Sbarro Health Research Organization, Temple University, Philadelphia, PA 19122 USA; 5 Venetian Institute of Molecular Medicine, University of Padova, Padova, Italy; 6 Department of Experimental Medicine, TOR, University of Rome Tor Vergata, 00133 Rome, Italy; 7 School of Computational and Integrative Sciences, Jawaharlal Nehru University, New Delhi, India; 8 Special Centre for Molecular Medicine, Jawaharlal Nehru University, New Delhi, India

**Keywords:** Epidemiology, Preclinical research

## Abstract

Coronavirus disease 2019 (COVID-19) is a severe acute respiratory syndrome caused by Coronavirus 2 (SARS-CoV-2). In the light of its rapid global spreading, on 11 March 2020, the World Health Organization has declared it a pandemic. Interestingly, the global spreading of the disease is not uniform, but has so far left some countries relatively less affected. The reason(s) for this anomalous behavior are not fully understood, but distinct hypotheses have been proposed. Here we discuss the plausibility of two of them: the universal vaccination with *Bacillus* Calmette–Guerin (BCG) and the widespread use of the antimalarial drug chloroquine (CQ). Both have been amply discussed in the recent literature with positive and negative conclusions: we felt that a comprehensive presentation of the data available on them would be useful. The analysis of data for countries with over 1000 reported COVID-19 cases has shown that the incidence and mortality were higher in countries in which BCG vaccination is either absent or has been discontinued, as compared with the countries with universal vaccination. We have performed a similar analysis of the data available for CQ, a widely used drug in the African continent and in other countries in which malaria is endemic; we discuss it here because CQ has been used as the drug to treat COVID-19 patients. Several African countries no longer recommend it officially for the fight against malaria, due to the development of resistance to *Plasmodium*, but its use across the continent is still diffuse. Taken together, the data in the literature have led to the suggestion of a possible inverse correlation between BCG immunization and COVID-19 disease incidence and severity.

## Introduction

The coronavirus disease 2019 (COVID-19) was first reported in Wuhan, China, in December 2019^1–3^. Since then, it has spread to most countries throughout the world, with major outbreaks in the United States, Spain, Italy, France, UK, Russia, China, South Korea, and Iran. On 11 March 2020, the World Health Organization (WHO) declared the severe acute respiratory syndrome caused by Coronavirus 2 (SARS-CoV-2) outbreak a global pandemic^4^. According to general consensus, SARS-CoV-2 originated from bats. There still is no conclusive indication on the transmitting animal host: somehow, the virus adapted to human hosts, rapidly spreading by human-to-human transmission^5–7^. As of today, there is still no vaccine that can protect against it. Specific anti-viral drugs that could treat the infection have been proposed and tested in clinical trials: the results have sometimes been promising, but not conclusive. The outcomes of the numerous randomized international clinical trials are about to appear and will hopefully provide conclusive answers. One of them has just appeared with positive results with remdesivir, an inhibitor of the RNA polymerase of COVID-19^8^.

The available data on COVID-19^9^ have revealed that the disease incidence and mortality vary, even dramatically, among countries. The variability could be due to a variety of factors such as ethnicity, dietary habits, climate, social activities, genetic differences, and governance structures. Here we would like to add to the list two potential factors that could possibly play a role in the susceptibility to the infection and in its gravity: the use of chloroquine (CQ) and the anti-tuberculosis (TB) vaccination (*Bacillus* Calmette–Guerin, BCG). The reason for considering them together in this contribution is that they are unique in their general presence/use in the populations which the COVID-19 pandemic has apparently so far only hit with minor violence. Our analysis of COVID-19 data from countries with universal BCG vaccination, discontinued vaccination, and countries that never adopted BCG vaccination suggests that disease incidence and morbidity are reduced in countries with universal BCG immunization compared with those that have not adopted the vaccine. The finding also applies to countries in which variables such as climate, dietary habits, and genetic origin essentially coincide. Suggestive examples are Spain and Portugal: the incidence of COVID-19 and its fatalities are at the time of writing this manuscript 386 per million population in Spain, where BCG vaccination has never been adopted, whereas, in the neighboring Portugal, in which general BCG vaccination was adopted in 1965, the disease incidence and mortality are 56 per million, respectively^9^. Further analysis of age-related susceptibility towards COVID-19 disease has revealed that countries with universal vaccination exhibit reduced disease incidence in all age groups. Countries that discontinued vaccination appear to exhibit disease incidence at levels intermediate between vaccinated and unvaccinated countries. There were significant differences in mortality in all age groups between the vaccinated and unvaccinated countries, although there were no differences between the unvaccinated countries and those that have discontinued vaccination.

The matter of CQ, a powerful regulator of endosomal pH in autophagy^10–13^ and cell death^14,15^, affecting distinct cell function^16,17^ and survival^18,19^, and of its derivative hydroxychloroquine (HCQ)^20^, has peculiar aspects: they are widely used in patients affected by the COVID-19 disease and reports from hospitals and doctors who treat patients frequently suggest beneficial effects. However, major medical institutions tend to recommend a cautious use, also pointing at dangerous side effects. In the absence of large-scale randomized trials, the effects on patients are frequently seen with significant doses of skepticism, which could also be motivated by the fact that HCQ is consistently used in late stages of the disease, when it would be too late to see its possible effects. This is an important point, because apart from their role in COVID-19 patients, which will be hopefully settled by the randomized trials now under way, here the presentation will focus on the possible chemoprophylactic properties of CQ and HCQ. It is common knowledge that shortages in their availability are recently being experienced in several countries that have not (yet) been hit by the full force of the pandemic. At the beginning of March, the Indian Council of Medical Research, under the Ministry of Health and Family Welfare, has recommended chemoprophylaxis with HCQ for asymptomatic healthcare workers who treat patients and for asymptomatic household contacts of confirmed COVD19 cases. It stated that its use in prophylaxis “is derived from available evidence of benefit as treatment and supported by preclinical data.” A recent large-scale international trial on HCQ sponsored by the WHO (the “Solidarity” trial) had produced negative results, prompting the WHO to interrupt it^21^. However, the trial itself has been severely criticized by a panel of 180 scientists and thus suggested to be invalid^22^. As a result of the criticism, WHO has actually decided to resume the trial.

## Results and discussion

The *Mycobacterium bovis* BCG strain was developed in 1921 at the Pasteur Institute through attenuation by serial passages of an *M. bovis* strain isolated from a cow with tubercular mastitis^23^. This strain was subsequently distributed to several laboratories in the world and a number of additional strains were then developed^24^. The six major BCG strains, namely Pasteur 1173 P2, Danish 1331, Glaxo 1077 (derived from the Danish strain), Tokyo 172-1, Russia BCG-I, and Moreau RDJ, now account for >90% of the BCG vaccines employed worldwide^25^. As five different strains of BCG were employed in different countries, we analyzed their correlative efficacy in protecting against COVID-19. Our data have shown that mixed and JAPAN strains seem superior to the DANISH strain. BCG exhibits efficacy against disseminated TB and meningitis in childhood^26,27^; currently, ~100 million children are vaccinated every year worldwide. However, the vaccine exhibits poor efficacy against adult pulmonary TB and several countries have, therefore, discontinued BCG immunization^23^. Subsequently, BCG was shown to exhibit protective effects against leprosy, buruli ulcer, and several other diseases, including those not associated with mycobacteria^28,29^. BCG is a potent immunomodulator, especially of the cell-mediated immunity, and is employed for the treatment of cancers such as that of the bladder^30^. It is actually being tested for several other cancers^31^. Recently, BCG was shown to exhibit efficacy against type I diabetes^32^. This was first shown in mice in Edmonton in 1993, which also partially protects against sepsis and respiratory infections when given following early infection^33^.

**Fig. 1:**
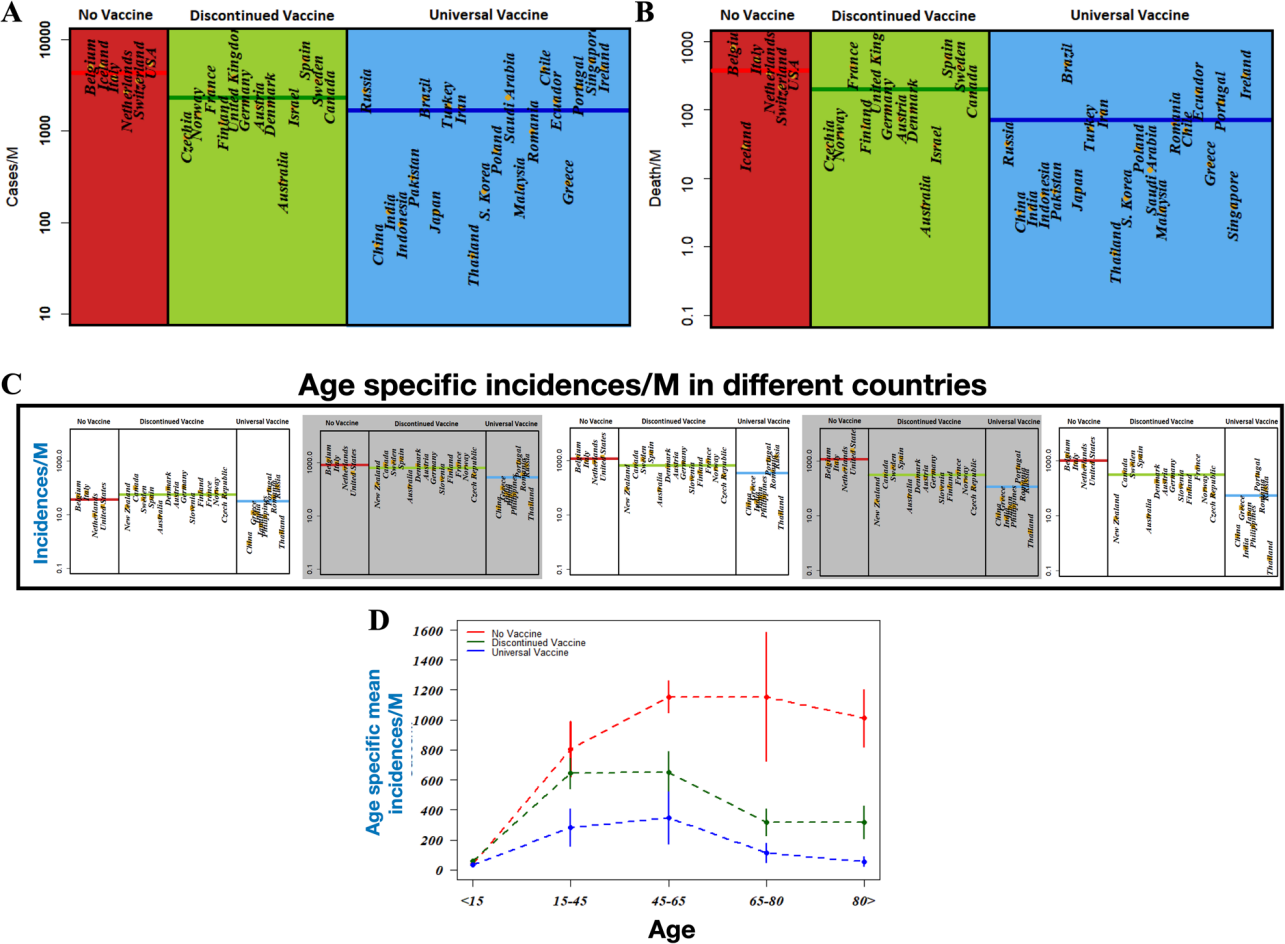
Impact of BCG immunization on incidences of COVID19. **a** and **b** represent the number of incident and deaths per million ofpopulation across countries employing continuous universal vaccination, some that have discontinued vaccination, and some that never adopted BCGvaccination. **c** Age specific incident cases per million of population for these three types of countries. **d** Age specific mean number of incident cases permillion along with standard deviations are presented for different types of countries.

To investigate the impact of BCG vaccination on the spread of COVID-19, we first classified countries into three groups following the data obtained from BCG ATLAS (“The BCG World Atlas” http://www.bcgatlas.org/index.php) till 29 May 2020. These three groups represent (i) countries that never adopted a national BCG vaccination program, (ii) countries that had a mass BCG vaccination program but discontinued it, and (iii) countries with an active national BCG immunization policy. We then extracted incidence and mortality of COVID-19 cases from the updated (up to 29 May 2020) data from the website of the Worldometer.info^9^. We selected countries with at least 1000 confirmed cases of COVID-19 (further details of the criteria for selection of countries are provided in the “Materials and Methods” section). The numbers of infections and deaths in these countries vary dramatically and cannot be compared directly, as the population sizes of these countries also vary (see Table 1). Therefore, we estimated the number of cases per million capita and presented the normalized measurements in Fig. 1 for the incidence and mortality, respectively. The results in Fig. 1a show that countries without a universal BCG policy (such as Belgium, Italy, the United States, and the Netherlands) have increased incidence of COVID-19 (2810.9 ± 497.1 (mean ± SEM) per million) compared with countries with ongoing national BCG policy (570.9 ± 155.6 (mean ± SEM) per million). The incidence for countries that discontinued BCG vaccination was intermediate between these two groups (1844.67 ± 508.89 (mean ± SEM) per million). In terms of morbidity, the countries with a universal BCG policy exhibited (see Fig. 1b) the lowest number of deaths due to COVID-19 (92.4 ± 34.7 (mean ± SEM) per million), which is significantly lower than that for countries that discontinued universal BCG vaccination (104.5 ± 33.6 (mean ± SEM) per million) (*p* = 0.001, Mann–Whitney *U*-test). Countries with no BCG vaccination were most profoundly affected (186.1 ± 56.8 (mean ± SEM) per million). These results are in line with two previous reports^34^.

**Table 1:** List of countries with total number of incident and death cases in COVID-19 as of 29 May 2020.

Universal BCG immunization				Discontinued universal BCG immunization			
Country	Population	Total cases	Total deaths	Country	Population	Total cases	Total deaths
				Germany	83,761,616	1,83,294	8600
China	1,439,323,776	83,001	4634	UK	67,855,909	272,826	38,376
India	1,378,826,256	182,990	5188	France	65,261,548	188,625	28,771
Indonesia	273,271,317	26,473	1613	Spain	46,753,295	286,308	27,125
Pakistan	220,497,647	69,496	1483	Canada	37,713,606	90,190	7073
Brazil	212,430,296	498,440	105,557	Australia	25,473,913	7195	103
Japan	126,507,477	16,804	886	Czechia	10,707,339	9233	319
Turkey	84,259,813	163,103	4515	Sweden	10,093,917	37,113	4395
Iran	83,897,889	151,466	7797	Israel	9,197,590	17,024	284
Thailand	69,785,445	3081	57	Austria	9,002,048	16,731	668
S. Korea	51,265,561	11,468	270	Denmark	5,790,499	11,633	571
Poland	37,849,973	23,686	1064	Finland	5,540,010	6859	320
Saudi Arabia	34,765,118	83,384	480	Norway	5,417,605	8437	236
Malaysia	32,329,329	7819	115	**Non-vaccinated**			
Romania	19,247,736	19,257	1262	**Country**	**Population**	**Total cases**	**Total deaths**
Chile	19,102,061	94,858	997	USA	330,838,184	1,816,897	1,05,557
Ecuador	17,619,020	38,571	3334	Italy	60,469,020	232,664	33,340
Greece	10,427,085	2915	175	Netherlands	17,131,732	46,257	5951
Portugal	10,199,093	32,203	1396	Belgium	11,585,389	58,381	9467
Singapore	5,846,395	34,884	23	Switzerland	8,649,211	30,862	1920
Ireland	4,932,957	24,929	1651	Iceland	341,055	1806	10

This type of analysis does not reflect the true impact of BCG against COVID-19. For example, a country with a higher percentage of population of elderly people may report more deaths than a country with a relatively younger population, simply because of increased comorbidities that worsen disease severity. Unfortunately, not enough public data are available to analyze the impact of such confounding factors. We therefore further probed how the incidence and mortality varies among different age groups in a population. We considered five different age groups, namely: <15, 15–44, 45–64, 64–79, and over 80 years of age. The results in Fig. 1c represent the distribution of disease incidence among different age groups for the three different types of countries. We also plotted the mean incidence of cases as a function of the different age groups for the three types of countries in Fig. 1d. The results indicate three significant features as follows: (i) the disease incidence is very low for subjects <15 years of age and does not show significant dependence on the presence or absence of universal BCG vaccination policies. This is possibly due to high resistance of young people to symptomatic disease. (ii) The number of infected cases across the age groups is always higher for countries without universal BCG vaccination policy. (iii) The differences between countries with universal BCG vaccine policy and countries without such a policy increase and reach their peak for age groups 45–64 and 65–79 years. The difference in the number of confirmed cases in these age groups is ~550–600 per million of the population between countries with vs. without universal BCG vaccination policy. The reduction in disease incidence seems significant for countries without universal BCG vaccination policy, where the number of confirmed cases per million is quite high for the younger age groups. The consistently reduced disease incidence across all age groups for countries with a universal BCG policy is in line with the suggestion of an inverse correlation between the BCG vaccine and COVID-19. In comparison, a rise in disease incidence across all age groups for countries without a universal BCG policy substantiates the hypothesis that BCG immunization could be repurposed to provide a weapon to combat the COVID-19 pandemic. In fact, very recently several countries, including the United States, Australia, Germany, and the Netherlands have initiated a BCG vaccination project aiming at the control COVID^35,36^. An intermediate level of protection for countries that have discontinued universal BCG vaccination policy further corroborates our claim.

**Fig. 2:**
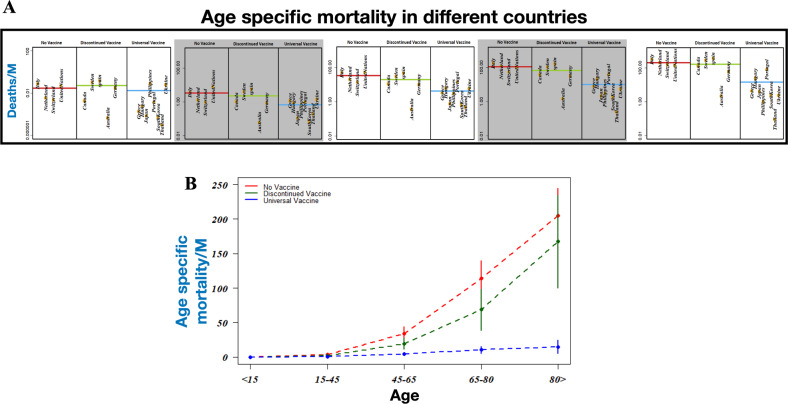
Impact of BCG immunization on death toll due to COVID19. **a** Age specific death cases per million of population for countries employing continuous universal vaccination, some that have discontinued vaccination, and some that never adopted BCG vaccination. **b** Age specific mean numberof death cases per million along with standard deviations are presented for different types of countries.

Having found that countries with universal BCG vaccination policy perform better in limiting the spread of COVID-19 compared with countries without a universal BCG vaccination or with countries that discontinued it, we next explored the association between BCG vaccination and COVID-19 mortality for different age groups. The result of our analysis is presented in the scattered plots of Fig. 2a, b, in which the mean number of deaths per million as a function of age groups is plotted for the three different types of countries. The results show that the distribution of fatal cases per million of people across different age groups for countries without mass vaccination and countries with discontinued mass vaccination are not significantly different (*p* = 0.41, Mann–Whitney *U*-test). With increasing age, the death toll increases for countries without or discontinued BCG vaccination programs. In comparison, the mortality is quite consistent and rises only slightly in the elderly population. Differences in the death toll between countries with or without a national BCG program thus suggest that BCG has a role in lowering the COVID-19 casualty by manifold. To verify the generality of our claim, we further compared our results based on the data collected till 6 April 2020 as presented in the Supplementary Information. The similarities between the previous results with the present one based on the data collected till 29 May 2020 supports our observation.

**Fig. 3:**
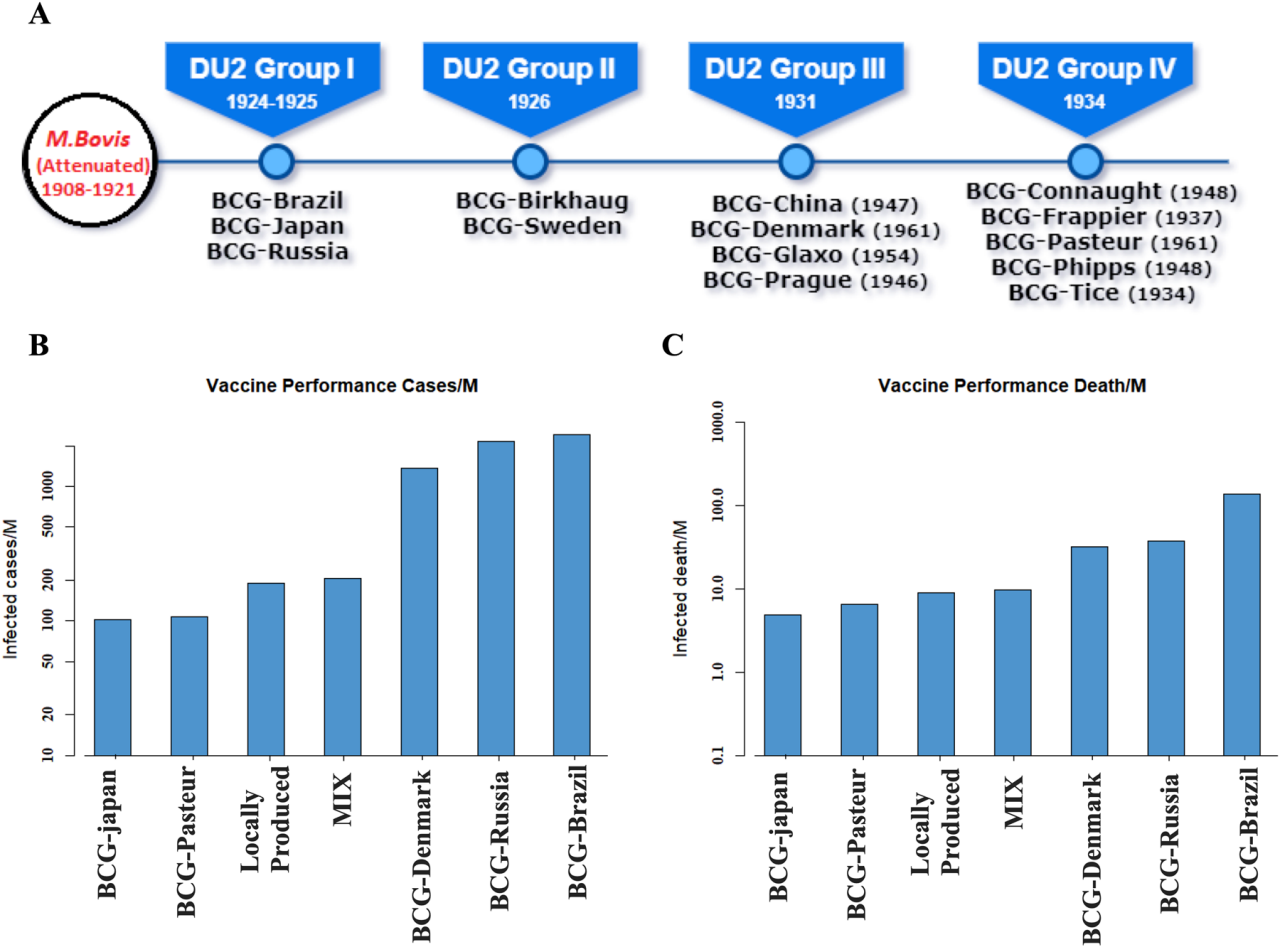
Efficacy of BCG strain against COVID19. **a** Types of BCG strain. **b** Number of COVID19 confirmed and **c** deaths cases observed forvarious BCG strains.

Although our results indicate a correlation between BCG vaccination and COVID-19 progression/mortality, one should also note that the confirmed cases to death ratio varies widely among the countries analyzed. This might be due to different strains of BCG used in these countries for an induced trained immunity. Figure 3a represents the evolution of several attenuated BCG strains and their timeline. To examine the potential effects of different BCG strains we considered seven strains, namely BCG-Russia, BCG-Denmark, BCG-Japan, BCG-Brazil, and BCG-Pasteur including a locally produced BCG strain and a mixed strain of BCG. We compared the number of confirmed cases and deaths per million that occurred within the populations that received different types of BCG vaccine. Figure 3b, c represent bar plots, indicating the incidence and mortality, respectively. We found that countries adopting a mixture of different strains of the BCG vaccine such as South Korea and the Philippines reported a lower number of confirmed and fatal cases. The BCG-Denmark and BCG-Russia strains correlated poorly in terms of limiting the COVID-19 spread and casualty. Taken together, the bulk of the data of the analysis appear to be compatible with a correlation between BCG vaccination and susceptibility to COVID-19 disease. To this end, a discussion related to certain countries, which have performed very differently compared with other countries. is essential One such example is Brazil. Even if it is a country with universal BCG vaccination policy, the number of deaths due to COVID-19 is the highest among the countries with similar vaccination strategy. This could be due to the use of the BCG-Brazil strain, which has been found ineffective against COVID-19 (see Fig. 3b). The case of Russia is probably similar, as the BCG strain used in Russia is probably also not effective against the coronavirus. A country that has done extremely well in restricting the COVID-19 spread is Australia. Although Australia has dropped its BCG program back in 1985, the number of incident and deaths due to COVID-19 are very low. This could be due to very low population density of Australia, which helped to restrict the spread of the virus effectively. However, caution on the BCG matter is still necessary: a recent report on a significant number of passengers of the Diamond Princess ship, who had recently become infected with COVID-19 has found no correlation^37^. Similarly, the BCG vaccine, which is routinely administered to all newborns in Israel and was administered to immigrants with unknown vaccination records, showed no statistically significant difference in the susceptibility to the COVID-19 infection^38^. Although these two studies seem to impose a strict cautionary note on the preventive use of BCG, it must be noted that the latter study had been performed only on a population of a specific age group in a country that had used an unknown BCG strain (“The BCG World Atlas” http://www.bcgatlas.org/index.php) in the period 1955–1982. The significance of the Israel study is thus difficult to evaluate, in particular as certain BCG strains are more effective against the COVID-19.

**Fig. 4:**
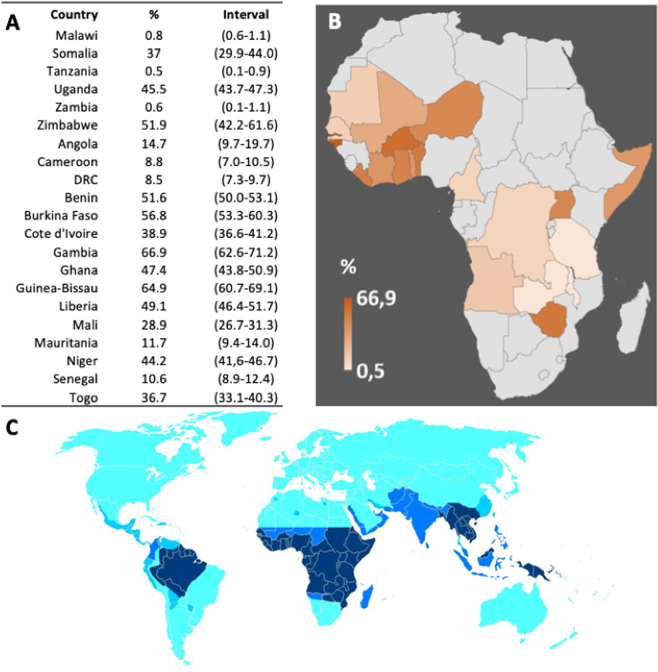
Distribution of Chloroquine use in African Countries. **a** Distribution of Chloroquine usage in African Countries, with interval ofconfidence. **b** Visualization of the data in panel A. **c** The map shows the occurrence of chloroquine resistance in the Plasmodium parasite, from darkblue (resistance at high levels) to light blue (no resistance). Countries in cyan are devoid of any malaria, but in those countries, chloroquine orhydroxychloroquine are still used for some autoimmune or inflammatory condition. Countries, where high chloroquine resistance occurs, are still among thehighest consumers of chloroquine, notwithstanding the adverse recommendation by most health authorities.

The possibility that the diffuse use of CQ and HCQ may possibly have a chemoprophylactic role in the populations that have so far been much less affected by the pandemic, e.g., those of most African countries or of India, could not be based on the accurate numerical analyses done for the BCG vaccination. However, although highly circumstantial, it should still be discussed, considering the still very diffuse consumption of CQ by populations in which malaria is still endemic. CQ has been instrumental in controlling the malaria tragedy, particularly its resurgence after the international ban on dichloro-diphenyl-trichloroethane, which had largely eliminated it by exterminating the *Anopheles* mosquito. Unfortunately, CQ therapy was compromised by the widespread appearance of drug resistance, particularly for *Plasmodium falciparum*: a threonine at position 76 of the CQ resistance gene *pfcrt* being the marker for CQ resistence^39^. As a result, following a recommendation of the WHO at the end of last century, most African countries officially discontinued its use in the fight against malaria, replacing it with artemisinin-based therapy. In a short time, however, the cessation of drug pressure led to the re-emergence of CQ-sensitive *Plasmodia*^40^: in Malawi, the first African country that had discontinued the use of CQ in 1993, by 2003 the mutant *pfcrt* gene was no longer present and CQ-sensitive malaria had regained predominance, as confirmed by the full efficacy of the drug in a clinical trial^41^. The pattern by which the resistance to CQ emerged and then declined had a strong regional character, with resistance emerging earlier in East Africa with respect to West Africa (see also Fig. 4). The pattern reflected the variation in the use of the drug but also reflected the degree of acceptance of the recommended official policy by the different African populations. The accurate analysis by Frosch et al.^40^. shows the consumption of CQ does not reflect the national policies of the African Countries. Although it varies among them, it has generally remained high (Fig. 4). Indirectly, this is also shown by recent contracts of pharmaceutical Companies to provide some African Countries with very large amounts of CQ and HCQ, and by the fact that some African Countries have initiated programs to produce them^34,42^. The situation is not limited to Africa: many countries outside Africa, including the United States, have reported short market supply of CQ and HCQ, and countries that produce them have banned their export. In India, the shortage of CQ has been attributed to widespread self chemoprophylaxis in the face of the COVID-19 pandemic^43^. However, caution should be taken^44^, as observational clinical trials for HCQ in COVID-19 patients was not associated with either a greatly lowered or an increased risk of the composite end point of intubation or death^45^ also in association with azithromycin^21,46^ and in view of potential toxicity as well^47^. On the other hand, as mentioned above, the negative results of the “Solidarity” international trial on HCQ^21^ have been indicated to be flawed. In any case, the “therapeutic” effect would not concern the discussion in this contribution, as we are considering its possible prophylactic role.

## Conclusions

In this study, we have presented the evidence available in the literature that has led to the suggestion of the possible effects of two factors, BCG vaccination and exposure to CQ, on the susceptibility to the COVID-19 infection. BCG vaccination is known to operate through the cell-mediated immunity that is important against COVID-19^48^. Although originally intended for tuberculosis, BCG immunization does not provide satisfactory results for the adult pulmonary disease, but confers partial protection against various other diseases^49^. We have presented the data, suggesting that the BCG immunization correlates with lower incidence and gravity of the COVID-19 disease across different countries, even when the BCG immunization was performed in childhood. Although the matter of CQ and HCQ does not have the degree of numerical sophistication of BCG vaccination, it still deserves to be considered. Unfortunately, the literature on CQ and HCQ has so far exclusively dealt with their therapeutic role in patients affected by the COVID-19 infection, frequently in its advanced stages. In this contribution, we have instead emphasized the possible chemoprophylactic role of CQ and HCQ: even if the evidence for it is admittedly only circumstantial, such a role should be tested in appropriately designed trials

## Materials and methods

To determine an association between BCG vaccination and COVID-19 disease incidence and mortality across several age groups of different countries, we first classified countries into three categories according to their respective BCG vaccination policy. These three classifications are as follows: countries that never adopted universal BCG vaccination, countries that adopted a universal BCG vaccination policy but subsequently discontinued it, and countries that currently exercise universal BCG vaccination^50^. The data for BCG vaccination policy across these countries were mapped using “The BCG World Atlas” (http://www.bcgatlas.org/index.php). As in a pandemic such as corona, constructing a data set at a single point in time may not be enough to capture its spread, we prepared two sets as follows: (i) data taken till 06 April and (ii) till 29 April 2020. The results based on the most updated data are presented in the manuscript, whereas similar analysis based on data till 06 April are given in the associated Supplementary Information. Our aim was to probe the number of incident and deaths due to COVID-19 in these three types of countries among different age groups. For this purpose, five age intervals were defined as the population below the age of 15, 15–44, 45–64, 65–79, and age above 80 years. Two types of comparative cross-sectional studies were performed as follows: (i) to compare the number of respective COVID-19 cases per million in the defined age groups of three defined classes of countries based on BCG policy; and (ii) to compare the number of respective COVID-19 deaths per million in the defined age groups of three defined classes of countries based on BCG policy. To obtain sufficient statistical power for data analysis, we only included countries with at least 1000 confirmed COVID-19 cases. As the number of cases reported in lower-income countries is very low and likely due to underreporting, we only included higher-income and middle-income countries as per the World Bank classification using the World Bank data (https://datahelpdesk.worldbank.org/knowledgebase/articles/906519-worldbank-country-and-lending-groups). We collected the coronavirus-related age-specific data of COVID-19 cases and deaths of countries from various sources (see Supplementary Materials). Two-tailed Mann–Whitney *U*-test was performed to test whether the two independent samples have the same distribution, where the null hypothesis *H*_0_ (i.e., both the samples are following the same distribution) was compared against an alternative hypothesis *H*_1_ (i.e., both the samples are following a different distribution), with a significance level of 0.05 (95% confidence interval). The significance of the test was governed by a *p*-value. A *p*-value below the level of significance (*p* < 0.05) indicates rejection of the null hypothesis^51^. Data were analyzed using R scripts.

## Supplementary information

BCG Vaccination Policy and preventive chloroquine usage: do they have an impact on COVID-19 pandemic?

Checklist
