# Supplementary material for: BCG vaccination policy and preventive chloroquine usage: do they have an impact on COVID-19 pandemic?
Source: Cell Death Dis. 2020 Jul 8;11(7):516. doi: 10.1038/s41419-020-2720-9 (PMC7341995; doi:10.1038/s41419-020-2720-9)
Supplement: Supplementary file 1 — BCG Vaccination Policy and preventive chloroquine usage: do they have an impact on COVID-19 pandemic? [file 41419_2020_2720_MOESM1_ESM.docx]

**CDDIS-20-1632R** **Supplementary Material**

BCG Vaccination Policy and preventive chloroquine usage: do they have an impact on COVID-19 pandemic?

Abhibhav Sharma^1^, Saurabh Kumar Sharma^1^, Jyoti Das^2^, Yufang Shi^3^, Enrico Bucci^4^, Ernesto Carafoli^5^, Gerry Melino^6^, Arnab Bhattacherjee^8^* and Gobardhan Das^7^*


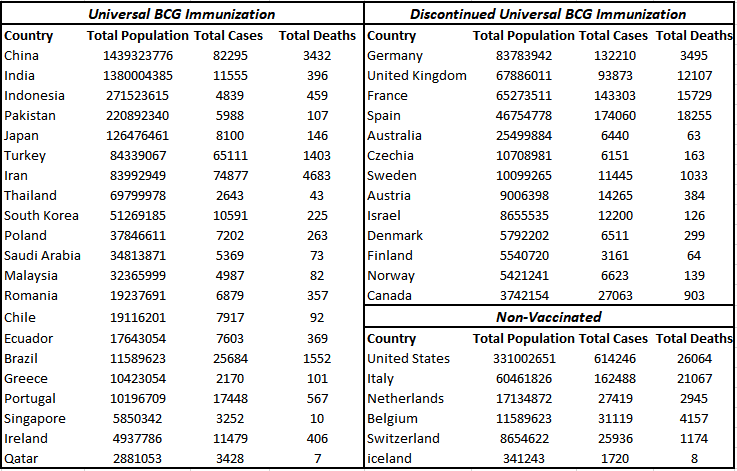


Table 1: List of countries with total number of incident and death cases in COVID 19 as of 14^th^ April 2020.

All analyses done below are based on data collected as on 06^th^ April 2020. We compared these results with the one presented in the main manuscript based on data collected till 29^th^ May 2020. The similarities between the results analysed based on datat collected at two different points of time supports our observation that BCG has a role in lowering the Covid-19 casualty by manifold.

**
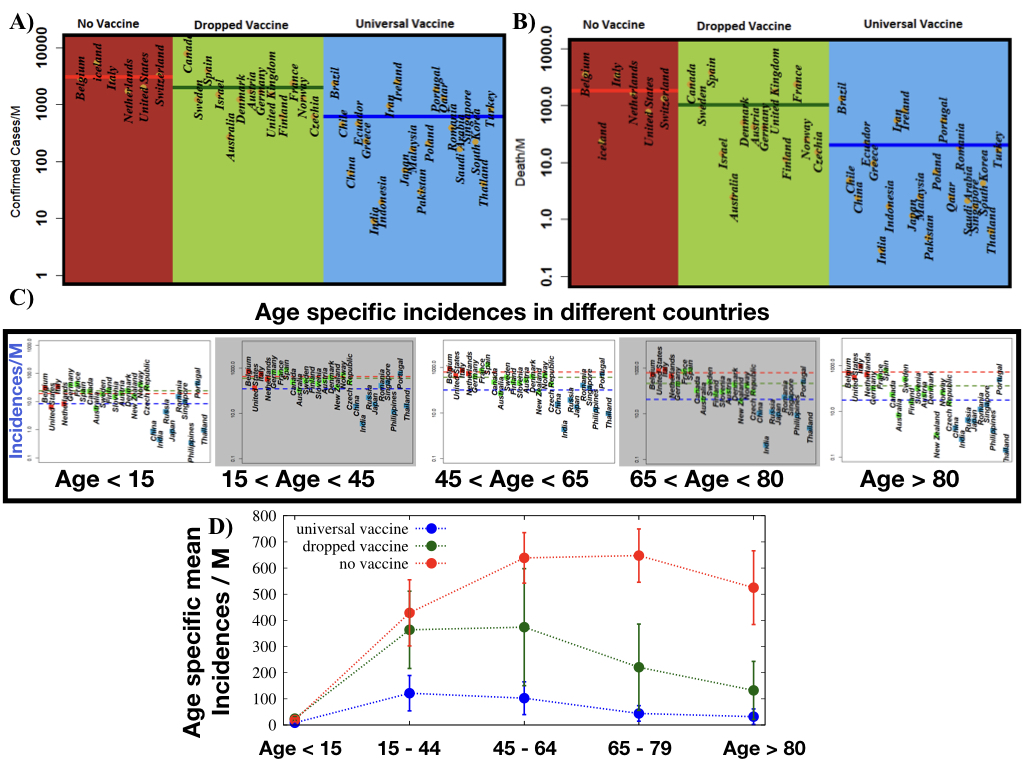
Figure 1: Impact of BCG immunization on incidences of COVID19 based on data as of 06^th^ April 2020**. (A) and (B) represent the number of incident and deaths per million of population across countries employing continuous universal vaccination, some that have discontinued vaccination, and some that never adopted BCG vaccination. (C) Age specific incident cases per million of population for these three types of countries. (D) Age specific mean number of incident cases per million along with standard deviations are presented for different types of countries.


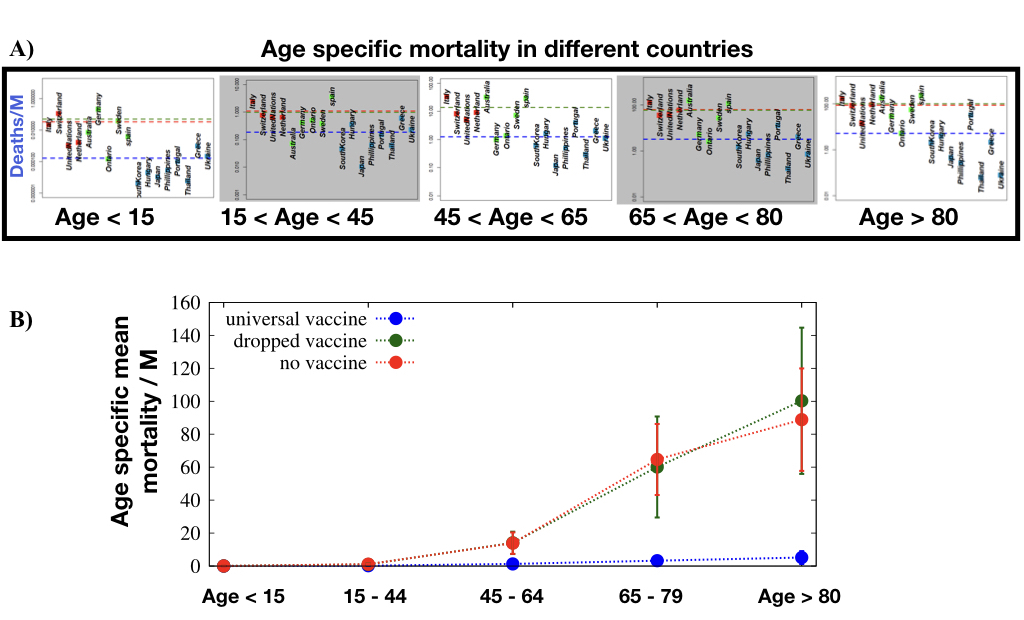


**Figure 2: Impact of BCG immunization on death toll due to COVID19** **based on data as of 06^h^ April 2020.** (A) Age specific death cases per million of population for countries employing continuous universal vaccination, some that have discontinued vaccination, and some that never adopted BCG vaccination. (B) Age specific mean number of death cases per million along with standard deviations are presented for different types of countries.


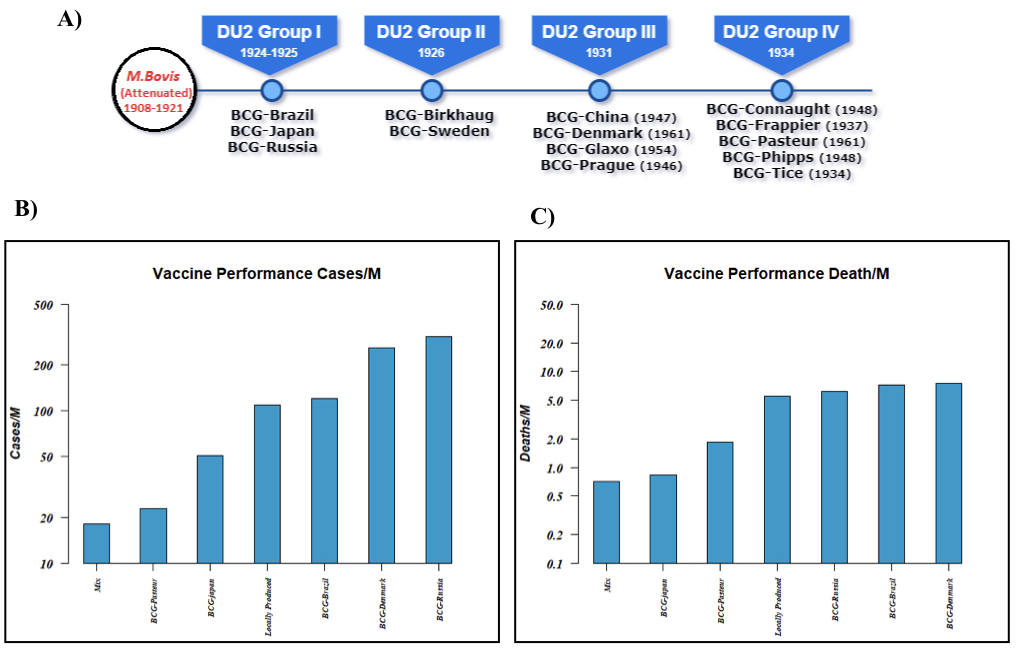


**Figure 3: Efficacy of BCG strain against COVID19 based on data as of 06^th^ April 2020**. (A) Types of BCG strain. (B) Number of COVID19 confirmed and (C) deaths cases observed for various BCG strains.
